# A multi-layer network model to assess school opening policies during a vaccination campaign: a case study on COVID-19 in France

**DOI:** 10.1007/s41109-022-00449-z

**Published:** 2022-03-07

**Authors:** Christian Bongiorno, Lorenzo Zino

**Affiliations:** 1 CentraleSupélec, Mathématiques et Informatique pour la Complexité et les Systèmes, Université Paris-Saclay, 91190 Gif-sur-Yvette, France; 2 Faculty of Science and Engineering, University of Groningen, 9747 AG Groningen, The Netherlands

**Keywords:** Complex networks, COVID-19, Epidemics, Multi-layer, Temporal network, Vaccination

## Abstract

We propose a multi-layer network model for the spread of an infectious disease that accounts for interactions within the family, between children in classes and schools, and casual contacts in the population. The proposed framework is designed to test several what-if scenarios on school openings during the vaccination campaigns, thereby assessing the safety of different policies, including testing practices in schools, diverse home-isolation policies, and targeted vaccination. We demonstrate the potentialities of our model by calibrating it on epidemiological and demographic data of the spring 2021 COVID-19 vaccination campaign in France. Specifically, we consider scenarios in which a fraction of the population is vaccinated, and we focus our analysis on the role of schools as drivers of the contagions and on the implementation of targeted intervention policies oriented to children and their families. We perform our analysis by means of a campaign of Monte Carlo simulations. Our findings suggest that transmission in schools may play a key role in the spreading of a disease. Interestingly, we show that children’s testing might be an important tool to flatten the epidemic curve, in particular when combined with enacting temporary online education for classes in which infected students are detected. Finally, we test a vaccination strategy that prioritizes the members of large families and we demonstrate its good performance. We believe that our modeling framework and our findings could be of help for public health authorities for planning their current and future interventions, as well as to increase preparedness for future epidemic outbreaks.

## Introduction

The ongoing COVID-19 pandemic has called for an unprecedented mobilization of the scientific community toward understanding the transmission mechanisms, designing and assessing non-pharmaceutical intervention policies (NPIs) to mitigate its spread, and developing effective vaccines. Within this joint effort, a forefront role has been played by the development of accurate mathematical models to predict the spread of the pandemic and assess the effectiveness of the implementation of different NPIs, such as wearing face masks, enforcing social distancing, and enacting travel bans (Estrada 2020; Vespignani 2020; Bertozzi et al. 2020). These works, grounded in the theory of mathematical modeling of epidemic diseases (Bailey 1975; Hethcote 2000) and on its recent application on complex networks (Pastor-Satorras et al. 2015; Mei et al. 2017; Nowzari et al. 2016; Paré et al. 2020; Zino and Cao 2021), have provided effective tools to assist public health authorities, by highlighting the epidemic risk, predicting its spatial and temporal spread, indicating limitations of the NPIs currently enacted, and suggesting potential strategies to improve them (Chinazzi 2020; Giordano et al. 2020; Gatto et al. 2020; Della Rossa et al. 2020; Parino et al. 2021; Arenas et al. 2020; Köhler et al. 2021; Carli et al. 2020).

From the inception of the epidemic outbreak, pharmaceutical researchers have started working at an unprecedented pace toward developing vaccines for the novel coronavirus SARS-CoV-2—which is the virus responsible for the COVID-19 disease—achieving the astonishing goal of developing, testing, and obtaining approval by national regulatory authorities for several vaccines in less than 1 year (https://ourworldindata.org/covid-vaccinations). Hence, in spring 2021, many countries around the world have started implementing an unprecedented vaccination campaign (https://ourworldindata.org/covid-vaccinations), while struggling with a second or third wave of the epidemic outbreak (World Health Organization 2020). Even in this phase, mathematical models have been valuable supports to assist public health authorities in their decisions on the vaccination strategies and on the policies that should be implemented during these phases (Grauer et al. 2020; Bubar et al. 2021; Truszkowska et al. 2021; Foy et al. 2021; Parino et al. 2021).

Among these questions, the policies concerning the management of schools and children play a crucial role for their impact on the education system and on their families (Gurdasani et al. 2021; Hyde 2020). Moreover, the impossibility of reducing contagions in schools by means of vaccinations—trials for vaccines on children started just as of February 2021 (https://www.nytimes.com/2021/02/12/health/covid-vaccines-children.htm)—makes crucial to understand how to calibrate NPIs in order to flatten the epidemic curve. For these reasons, understanding the effect of different policies for school opening, including increasing the testing rate for children and implementing temporary online education in order to home-isolate the entire class whenever a child is tested positive in that class, is a problem of paramount importance. We also believe that similar issues might emerge again in the case of future pandemics, since new drugs and vaccines are typically initially tested on adult individuals, while trials on children start at a later stage. Hence, developing a mathematical framework to assess the safety of school opening policies and test what-if scenarios focused on the role of children in the spread of infectious diseases might be key not only to face the current problems, but also to create preparedness against future threats.

Motivated by these important public health problems, we propose a temporal network modeling framework (Holme and Saramäki 2012; Holme 2015) with a multi-layer structure (Kivelä et al. 2014), tailored to capture the spreading of airborne diseases, with a specific focus on the transmission between children in schools and in their families. Specifically, the proposed model is developed on four layers: a *family layer*, which represents the interactions between family members living in the same household; a *class layer* that models the interactions between children in the same class, a *school building layer* that represents the interactions between students of different classes that are placed in the same building (e.g., when entering/exiting the building or during sports activities or class breaks); and a *contact layer*, which captures casual interactions between adults, for instance, in shops or in public transport. While the family layer is assumed to be fixed, the other three layers are time-varying, allowing to capture different phenomena, including the implementation of home-isolation policies. In particular, while we assume that students’ membership to classes does not change in the time-horizon of our modeling framework, the interactions between students that belong to the same class may change if students—or even entire classes—are home-isolated. The time-varying school building layer and the contact layer, instead, are generated in a stochastic fashion, representing casual contacts between students of different classes and between adults that are not home-isolated, respectively. These stochastic time-varying interactions are generated using an activity-driven network model (Perra et al. 2012; Zino et al. 2016), which has emerged as a valuable modeling framework to generate heterogeneous time-varying networks of interactions (Starnini and Pastor-Satorras 2013).

The network model is combined with a generic and flexible compartmental model for the disease progression that captures key features common to most airborne diseases (Pastor-Satorras et al. 2015). Specifically, the proposed model encapsulates a latency period, partial detection of infected individuals, and vaccinations. Furthermore, the disease progression model takes into account the inherent differences in infectiousness and in the emergence of symptoms (and thus in the detectability) between children and adults. To this aim, we consider two distinct classes of individuals—children and adults—each one characterized by different parameters for the infection probability and the detection rate. It worth commenting that both the network model and the compartmental model can be easily extended by including further features (e.g., an age-stratification structure or further compartments), or incorporated as a component of a larger modeling framework (as often happens with agent-based models).

The model is demonstrated in a scenario inspired by the COVID-19 pandemic. The network model is calibrated by generating a population corresponding to a small-medium size urban area, with a demographic distribution calibrated to the French demographic data (https://www.insee.fr/fr/statistiques/4277630?sommaire=4318291&fbclid=IwAR3z-EUWTcRXgeE5VK-XE3Mkk6SugqJXZG1ox4r0qi7tRo220DpvLErRKvY, https://www.education.gouv.fr/les-chiffres-cles-du-systeme-educatif-6515), and by tailoring the epidemic progression model to COVID-19, by calibrating the epidemic parameters utilizing reliable estimations from the epidemiology literature (Prem 2020; Zhang et al. 2020; Davies et al. 2021; Dattner et al. 2021). We use the calibrated model to investigate the role of children and schools in the spreading of COVID-19 and to perform what-if analyses toward assessing the effectiveness of different policies during the spring 2021 vaccination campaign. Our analyses, performed through an extensive campaign of Monte Carlo simulations, allow us to draw some conclusions. First, we use the model to support the intuition that children play a key role in the spreading of COVID-19, and thus—being the vaccination of children still not viable (https://www.nytimes.com/2021/02/12/health/covid-vaccines-children.htm)—the management of NPIs in schools seems crucial to keep the infections under control while vaccinating the adults. Second, massive testing campaigns in schools seem to be effective in mitigating the spread. However, these campaigns may be practically unfeasible, since they may require detecting at least 70% of the infections (including asymptomatic) to be able to flatten the curve—an objective that might be far beyond the current estimates (Pullano et al. 2020). Third, the enforcement of online education for schoolmates of detected infected children seems to be an effective practice to keep the number of infections under control, in combination with a moderate testing campaign in schools. Finally, we find that prioritizing vaccination of large families may be a valuable strategy to reach herd immunity faster, limiting the need for massive testing campaigns in schools. We believe that these findings might be of help to assist public health authorities during the next phases of the fight against COVID-19. Moreover, the generality of our modeling framework and its flexibility suggests that it could be a valuable tool to investigate issues that may arise in the future stages of the pandemic (e.g., due to the spread of new highly-contagious strains), and to increase preparedness for future airborne epidemic outbreaks.

In summary, the main contribution of this paper is twofold. First, we extend the literature on networks epidemic models by proposing a well-grounded agent-based method for the study of the interplay of an airborne epidemic disease and human decisions. This method allows for accurately reproduce the social structure that underlies disease transmission in communities with families, schools, and other social interactions, and enables the assessment of different control strategies to mitigate the spread. Second, we adopt the proposed model to study a case study calibrated on the vaccination campaign during the COVID-19 pandemics, gaining novel insights that could be of interest to help assist public health authorities during the current and future phases of the COVID-19 pandemics.

The rest of the paper is organized as follows. In “Model” section, we present our multi-layer network epidemic model. In “Model calibration to COVID-19 in France and simulation setting” section, we calibrate our general modeling framework to investigate the ongoing COVID-19 challenges. In “Results” section, we present our main results and discuss their implications. “Discussion and conclusions” section concludes the paper by summarizing the take-home message and outlining future research directions.

## Model

### Population

We consider a population of *n* individuals, $n\in {\mathbb {Z}}^+$, indexed by ${\mathcal {V}}=\{1,\ldots ,n\}$, where ${\mathbb {Z}}^+$ is the set of non-negative integer numbers. Individuals are divided into two types: *children* ${\mathcal {C}}=\{1,\ldots ,{\tilde{n}}\}$ and *adults* ${\mathcal {A}}=\{{\tilde{n}}+1,\ldots ,n\}$. The entire population is partitioned into a set ${\mathcal {F}}=\{{\mathcal {F}}_1,\ldots {\mathcal {F}}_k\}$ of *k* mutually exclusive families (so that ${\mathcal {V}}=\bigcup _{\ell =1}^k F_\ell$). Similarly, children are partitioned into a set ${\mathcal {S}}=\{{\mathcal {S}}_1,\ldots {\mathcal {S}}_m\}$ of *m* mutually exclusive school classes (so that ${\mathcal {C}}=\bigcup _{\ell =1}^m{\mathcal {S}}_\ell$). In a hierarchical fashion, children are also assigned to a set of *p* mutually exclusive school buildings ${\mathcal {B}}=\{{\mathcal {B}}_1,\ldots {\mathcal {B}}_p\}$ (so that ${\mathcal {C}}=\bigcup _{\ell =1}^p{\mathcal {B}}_\ell$). We assume that the population and its partitioning in families and school classes remain constant throughout the duration of the epidemic outbreak.

**Fig. 1:**
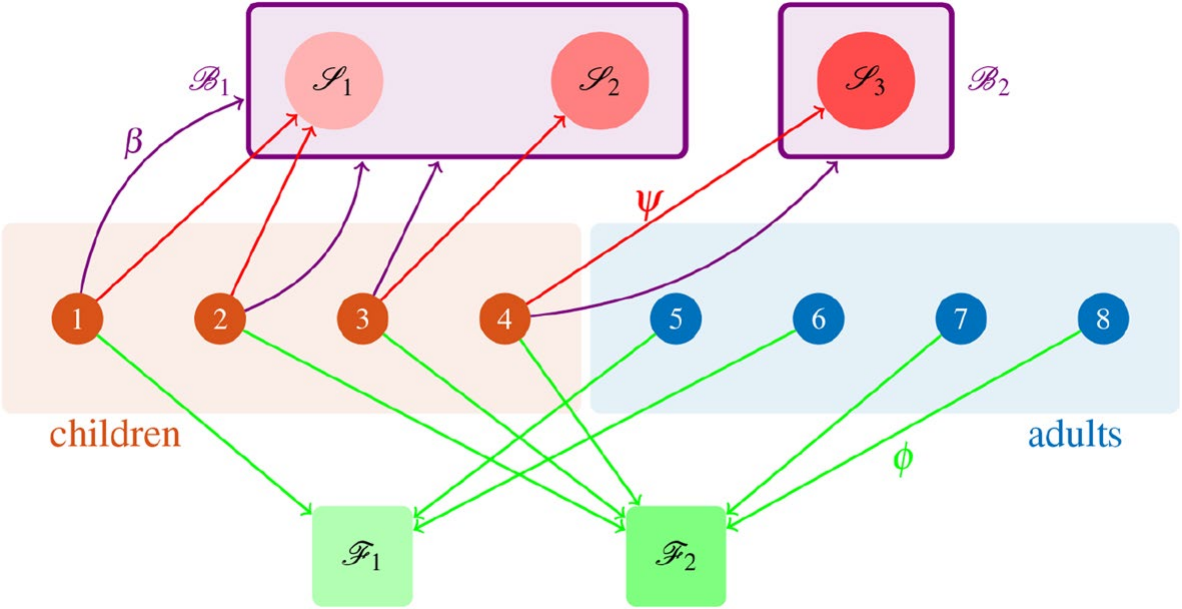
Schematic of the population structure. In this simple example, four children ${\mathcal {C}}=\{1,2,3,4\}$ and four adults ${\mathcal {A}}=\{5,6,7,8\}$ are partitioned into three school classes (red circles above), placed into two different buildings (violet rectangles), and two families (green squares below), by means of the functions $\psi$ and $\phi$

We define three functions $\phi$, $\psi$, and $\beta$ that associate each individual with their family, and each child with their class and school building, respectively. Specifically, we denote by $\phi :{\mathcal {V}}\rightarrow {\mathcal {F}}$ the function that associates each individual with the corresponding family; by $\psi :{\mathcal {C}}\rightarrow {\mathcal {S}}$ the function that associates each child with their class; and by $\beta :{\mathcal {C}}\rightarrow {\mathcal {B}}$ the function that associates each child with the school building in which their class is. Clearly, due to the hierarchical structure, if $\psi (i)=\psi (j)$, then necessarily $\beta (i)=\beta (j)$. Hence, each adult $i\in {\mathcal {A}}$ is associated with their family $\phi (i)$, while each child $j\in {\mathcal {C}}$ is associated with their family $\phi (j)$, class $\psi (j)$, and building $\beta (j)$. A schematic of the population structure is illustrated in Fig. 1.

### Network

Most airborne diseases spread through human-to-human interactions between individuals. In this section, we introduce a time-varying network structure such that the presence of a link between a pair of individuals at a certain time means that the two individuals have an interaction at that time (of duration sufficiently long to allow contagion). Specifically, we define a time-varying multi-layer network structure (Kivelä et al. 2014), which accounts for the presence of steady interactions within each family, intermittent interactions in classes, and casual time-varying contacts (Holme and Saramäki 2012; Holme 2015). To this aim, we propose a four-layered undirected network structure ${\mathcal {G}}=({{\mathcal {V}},{\mathcal {E}}_{F},{\mathcal {E}}_S(t),{\mathcal {E}}_B(t),{\mathcal {E}}_C(t)})$, $t\in {\mathbb {Z}}^+$, in which the four layers capture the interactions between family members living in the same household, between classmates, between children in different classes, and other casual contacts, respectively. Note that the edge set ${\mathcal {E}}_F$ is assumed to be constant, while the other three layers are time-varying, reflecting the assumption that—at least on a time-scale relative to an epidemic outbreak—families do not change, while the implementation of online education may reduce the physical interactions between classmates, and the pattern of casual contacts in school buildings and between adults may vary day-to-day. The four layers are defined as follows.

**Family layer**. The family layer is defined by the time-invariant edge set ${\mathcal {E}}_{F}\subseteq {\mathcal {V}}\times {\mathcal {V}}$, which captures the interactions between family members, that is,

1 $$\begin{aligned} (i,j)\in {\mathcal {E}}_F\iff \phi (i)=\phi (j)\,. \end{aligned}$$

Hence, the family layer is formed by a set of cliques, connecting all the individuals in the same family ${\mathcal {F}}_\ell$. This is based on the assumption that all the individuals that share a room or an indoor environment with an infected individual for several hours per-day (e.g., in the household) have a non-negligible probability of being infected (Bazant and Bush 2021; Lewis 2021).

**Class layer**. The class layer is defined by a time-varying edge set ${\mathcal {E}}_{S}(t)$, which represents the interactions between classmates at time *t*. Formally, for each children $i\in {\mathcal {C}}$, we define a variable $A_i(t)\in \{0,1\}$ termed *home-isolation state* representing whether *i* goes to school at time *t* ($A_i(t)=1$) or if they stay at home ($A_i(t)=0$). Note that children can stay at home either for periodic school closures (for instance, during weekends) and for the implementation of NPIs (for instance, by enforcing online education for infected children, or for their entire class). The interactions within the classes at time *t* are then defined as follows:

2 $$\begin{aligned} (i,j)\in {\mathcal {E}}_S(t)\iff \psi (i)=\psi (j)\quad \text {and}\quad A_i(t)=A_j(t)=1\,. \end{aligned}$$

Similar to the family layer, also the class layer is made of cliques, since all the children that share a class for several hours with an infected individual may be infected.

**Fig. 2:**
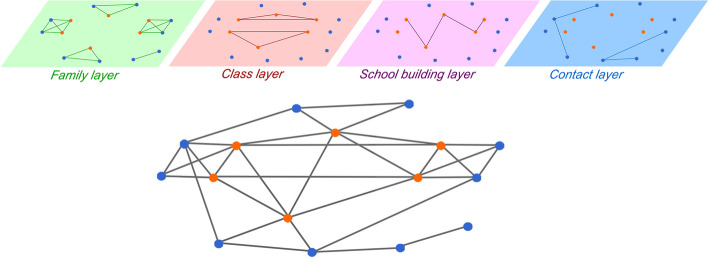
Schematic of the four-layered network structure with a sample realization of the four distinct layers in a time-step *t* (top), and of the corresponding aggregate network of interactions (bottom). Orange nodes represent children (which are partitioned into two classes in the same building), blue nodes represent adults

**School building layer**. The school building layer is defined by a time-varying edge set ${\mathcal {E}}_{B}(t)$, which represents the interactions between children in different classes but in the same building at time *t*, which may occur in the corridors, when entering and exiting the building or during class breaks. We generate the random interactions between children in different classes ${\mathcal {E}}_{B}(t)$ by using an extension of a discrete-time activity-driven network (ADN) (Perra et al. 2012). We adopt ADNs—which have emerged as a powerful modeling framework to realistically reproduce time-varying heterogeneous networks (Starnini and Pastor-Satorras 2013)—because of their flexibility (Rizzo et al. 2014; Moinet et al. 2015; Nadini et al. 2018; Bongiorno et al. 2019) which allows us to incorporate the specific features of our model such as different home-isolation policies, and for their amenability to efficiently perform fast numerical simulations (Rizzo et al. 2016). Similar to a standard ADN (Perra et al. 2012), each child is characterized by a constant parameter $a_i\in [0,1]$, $i\in {\mathcal {C}}$, called *activity*, expressing their propensity to interact with others; all the children have a common parameter $m_c\in {\mathbb {Z}}_{>0}$, which represents the number of interactions that active children initiate. Then we define the following algorithm to generate the school building layer ${\mathcal {E}}_{B}(t)$, at each time step *t*, independently of the previous time steps:

1. The edge set is initialized as an empty edge set ${\mathcal {E}}_B(t)=\emptyset$;
2. Each child $i\in {\mathcal {C}}$ that is not home isolated ($A_i(t)=1$) activates with probability equal to $a_i$, independent of the others and of the previous history of the process;
3. If *i* activates, then the children generates $m_c$ undirected links with a $m_c$-tuple of (non-home-isolated) children outside their class ($\psi (i)$) but in the same building ($\beta (i)$), selected uniformly at random in the set $\{j:j\in {\mathcal {C}}, \psi (j)\ne \psi (i), \beta (j)=\beta (i), A_j(t)=1\}$; the generated links are added to the set ${\mathcal {E}}_B(t)$;

**Contact layer**. The contact layer is defined by the time-varying edge set ${\mathcal {E}}_C(t)\subseteq {\mathcal {A}}\times {\mathcal {A}}$, representing the casual interactions that are generated, for instance, in a shop, or in public transportation, or at work. We assume that only adults individuals are involved in this type of interaction, while children only interact with their family members and their classmates. This is consistent with the presence of mobility restrictions and with the closure of most non-essential activities during the early vaccination stages. Similar to children, also each adult is associated with a *home-isolation state* $A_i(t)\in \{0,1\}$, $i\in {\mathcal {A}}$, representing whether *i* is allowed to have social interactions at time *t* ($A_i(t)=1$), or if they are home-isolated ($A_i(t)=0$). Similar to the school building layer, we generate these random interactions by using an extension of a discrete-time activity-driven network (ADN) (Perra et al. 2012). Specifically, we associate to each adult their activity $a_i\in [0,1]$, $i\in {\mathcal {A}}$ and we fix a common parameter $m_a\in {\mathbb {Z}}_{>0}$. Then, at each time step *t* (and independently of the previous time steps), the contact layer is defined as follows:

1. The edge set is initialized as an empty edge set ${\mathcal {E}}_C(t)=\emptyset$;
2. Each adult $i\in {\mathcal {A}}$ that is not home isolated ($A_i(t)=1$) activates with probability equal to $a_i$, independent of the others and of the previous history of the process;
3. If *i* activates, then the adult generates $m_a$ undirected links with a $m_a$-tuple of (non-home-isolated) adults outside their family ($\phi (i)$), selected uniformly at random in the set $\{j:j\in {\mathcal {A}}, \phi (j)\ne \phi (i), A_j(t)=1\}$; the generated links are added to the set ${\mathcal {E}}_C(t)$;

The multi-layer network structure obtained is illustrated in Fig. 2.

#### **Remark 1**

This multi-layer structure can be extended to incorporate further real-world features, which have been proven to play a key role in the spread of epidemics (Rader et al. 2020). For instance, workplaces for adults can be modeled in a hierarchical structure with offices and buildings, similar to classes and schools; and households can be placed in neighborhoods. All these extensions can be implemented by simply adding layers, without changing the very fabric of our modeling paradigm.

### Disease progression and spreading

**Fig. 3:**
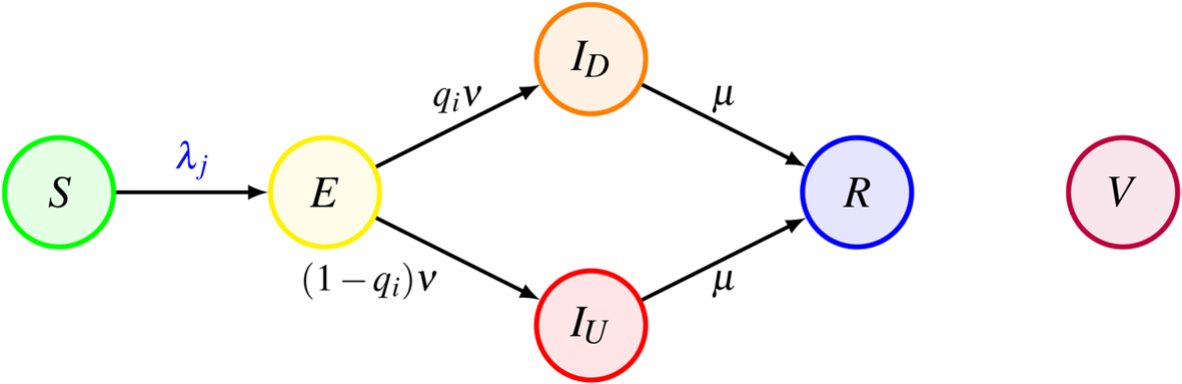
State transitions characterizing the epidemic spreading model. Susceptible individuals (*S*) that have interactions with infectious individuals ($I_D$ and $I_U$) may become exposed (*E*), and then infectious. Infectious individuals can be either detected ($I_D$) or unaware ($I_U$). Then, they may recover or die, becoming removed (*R*). Vaccinated individuals cannot contract the disease

We consider an extension of a stochastic network susceptible–exposed–infectious–removed (SEIR) model on networks, which captures several important features of common airborne diseases (Pastor-Satorras et al. 2015; Zino et al. 2017). In this model, we include two additional compartments to account for vaccinations and for the presence of asymptomatic unaware infectious individuals. Specifically, at discrete time instant $t\in {\mathbb {Z}}^+$, each individual $i\in {\mathcal {V}}$ is characterized by a variable $X_i(t)\in \{S,E,I_D,I_U,R,V\}$, representing the *health state* of the individual at time *t*. The state *S* represents *susceptible* individuals, who are healthy and can be infected by the disease, while individuals that have been vaccinated and are immune to the disease are denoted by *V*. Susceptible individuals who have been *exposed* to the disease and are thus infected, but not yet infectious, are represented by *E*. Infectious individuals are divided into two compartments: those that are detected (due to the emergence of symptoms or because of receiving a positive test), denoted by $I_D$, and those that are asymptomatic and not tested, and thus unaware ($I_U$). Finally, individuals that recover (or die) are denoted by *R*. We assume that recovered individuals become immune to the disease. The progression of the disease is described in the following and illustrated in Fig. 3.

At each time-step *t*, susceptible individuals ($X_i(t)=S$) may enter in contact with the pathogen due to interactions with infectious individuals ($I_D$ and $I_U$) and thus they may become exposed ($X_i(t+1)=E$). The *per-contact infection probability* $\lambda _j\in [0,1]$, is a constant parameter that captures the probability that the disease is transmitted through a physical contact with an infectious individual *j*, and may differ depending on the individual *j*. Specifically, we assume that children and adults may have different contagion probabilities. Hence, we introduce two parameters $\lambda _a\in [0,1]$ and $\lambda _c\in [0,1]$ to model the *adults per-contact infection probability* and the *children per-contact infection probability*, respectively, and we set

3 $$\begin{aligned} \lambda _j=\left\{ \begin{array}{ll} \lambda _c&{}\quad \text {if}\quad j\in {\mathcal {C}},\\ \lambda _a&{}\quad \text {if}\quad j\in {\mathcal {A}}. \end{array}\right. \end{aligned}$$

Hence, the contagion probability for each individual $i\in {\mathcal {V}}$ is equal to

4 $$\begin{aligned} {\mathbb {P}}[X_i(t+1)=E|X_i(t)=S]=1-(1-\lambda _c)^{N^{F,c}_i(t) +N^S_i(t)}(1-\lambda _a)^{N^{F,a}_i(t)+N^C_i(t)}\,, \end{aligned}$$

where, denoted by $|\cdot |$ the cardinality of a set,

5a $$\begin{aligned}&N^{F,c}_i(t)=|\{j\in {\mathcal {C}}:(i,j)\in {\mathcal {E}}_F,\,X_j(t) \in \{I_U,I_D\}\}|, \end{aligned}$$

5b $$\begin{aligned}&N^{F,a}_i(t)=|\{j\in {\mathcal {A}}:(i,j)\in {\mathcal {E}}_F,\,X_j(t) \in \{I_U,I_D\}\}|,\end{aligned}$$

5c $$\begin{aligned}&N^S_i(t)=|\{j{\in {\mathcal {C}}}:(i,j)\in {\mathcal {E}}_C(t) \cup {\mathcal {E}}_S(t),\,X_j(t)\in \{I_U,I_D\}\}|,\end{aligned}$$

5d $$\begin{aligned}&N^C_i(t)=|\{j{\in {\mathcal {A}}}:(i,j)\in {\mathcal {E}}_C(t), \,X_j(t)\in \{I_U,I_D\}\}|, \end{aligned}$$

are the number of infectious individuals that have a link with individual *i* at time *t* on the family (children and adults, separately), class and school building, and contact layer, respectively. Note that, while the interactions on the family layer are split into two sets $N^{F,c}_i(t)$ and $N^{F,a}_i(t)$ to separately account for children and adults (since in general they may have different per-contact infection probability), the interactions on the school buildings and class layers involve only children ($N^S_i(t)\subseteq {\mathcal {C}}$) and those on the contact layer involves only adults ($N^C_i(t)\subseteq {\mathcal {A}}$).

Besides the contagion, at each time-step *t*, each exposed individual *E* may become infectious, with probability $\nu \in [0,1]$. Infectious individuals may be aware ($I_D$) due to being symptomatic or receiving a positive test, or unaware ($I_U$) due to being asymptomatic and untested. We denote by $q_i\in [0,1]$ the probability that individual *i* is detected if infected. We assume that such a probability depends on whether the individual is a children or an adult, as a consequence of different probabilities of developing symptoms and different testing policies that can be implemented for the two types of individuals. Hence, we introduce two parameters $q_a\in [0,1]$ and $q_c\in [0,1]$ to model the *adults detection rate* and the *children detection rate*, respectively, and we set

6 $$\begin{aligned} q_i=\left\{ \begin{array}{ll} q_c&{}\quad \text {if}\quad i\in {\mathcal {C}},\\ q_a&{}\quad \text {if}\quad i\in {\mathcal {A}}. \end{array}\right. \end{aligned}$$

Infectious individuals recover or die and become removed (*R*), with probability $\mu \in [0, 1]$. These transitions, illustrated in Fig. 3, are thus governed by the following probabilistic rules:

7a $$\begin{aligned}&{\mathbb {P}}[X_i(t+1)=I_D\,|\,X_i(t)=E]=q_i\nu \,, \end{aligned}$$

7b $$\begin{aligned}&{\mathbb {P}}[X_i(t+1)=I_U\,|\,X_i(t)=E]=(1-q_i)\nu \,,\end{aligned}$$

7c $$\begin{aligned}&{\mathbb {P}}[X_i(t+1)=R\,|\,X_i(t)\in \{I_D,I_U\}]=\mu \,. \end{aligned}$$

Table 1 summarizes the notation use throughout this paper.

**Table 1:** Notation used in the paper

Notation	Meaning
${\mathcal {V}}=\{1,\ldots ,n\}$	Population
${\mathcal {C}}=\{1,\ldots ,{\tilde{n}}\}$	Children
${\mathcal {A}}=\{{\tilde{n}}+1,\ldots ,n\}$	Adults
${\mathcal {F}}=\{{\mathcal {F}}_1,\ldots ,{\mathcal {F}}_k\}$	Families
${\mathcal {S}}=\{{\mathcal {S}}_1,\ldots ,{\mathcal {S}}_m\}$	School classes
${\mathcal {B}}=\{{\mathcal {B}}_1,\ldots ,{\mathcal {B}}_p\}$	School buildings
$\phi :{\mathcal {V}}\rightarrow {\mathcal {F}}$	Function that associates individuals with their families
$\psi :{\mathcal {C}}\rightarrow {\mathcal {S}}$	Function that associates children with their classes
$\beta :{\mathcal {C}}\rightarrow {\mathcal {S}}$	Function that associates children with their buildings
${\mathcal {E}}_F\subseteq {\mathcal {V}}\times {\mathcal {V}}$	Family layer
${{\mathcal {E}}_S}(t)\subseteq {\mathcal {C}}\times {\mathcal {C}}$	Class layer at time *t*
${{\mathcal {E}}}_B(t)\subseteq {\mathcal {C}}\times {\mathcal {C}}$	School building layer at time *t*
${\mathcal {E}}_C(t) \subseteq {\mathcal {A}}\times {\mathcal {A}}$	Contact layer at time *t*
$a_i\in [0,1]$	Activity of individual *i*
$m_c\in {{\mathbb {Z}}_{>0}}$	Interactions initiated by an active child
$m_a\in {{\mathbb {Z}}_{>0}}$	Interactions initiated by an active adult
$X_i(t)\in \{S,E,I_D,I_U,R,V\}$	Health state of individual *i* at time *t*
$A_i(t)\in \{0,1\}$	Home-isolation state of individual *i* at time *t*
$\lambda _a\in [0,1]$	Adult per-contact infection probability
$\lambda _c\in [0,1]$	Children per-contact infection probability
$\nu \in [0,1]$	Probability of becoming infectious
$\mu \in [0,1]$	Recovery probability
$q_a\in [0,1]$	Adults detection rate
$q_c\in [0,1]$	Children detection rate

#### **Remark 2**

The disease progression model is amenable to several extensions, to incorporate further features of real-world epidemics. For instance, further compartments and transition probabilities can be included in the model to account for the different stages and possibility of treatment (e.g., in standard hospital beds or in intensive care unit), the possible implementation of quarantine, to differentiate the possible outcomes of the disease (e.g., recovery or death), and to account for possible reinfections. See, e.g., the recent models developed to capture the disease progression of COVID-19 (Estrada 2020; Giordano et al. 2020).

#### **Remark 3**

The contagion probability in (4) can be expanded by utilizing location-dependent per-contact infection probabilities, as it is often assumed in agent-based models (Chinazzi 2020; Truszkowska et al. 2021), so that interactions in different places may be associated with different transmission probabilities (e.g., to model indoor vs. outdoor locations or the use of personal protective equipment).

### Dynamics

Under the reasonable assumption that whether an individual is home-isolated at time *t* depends only on the health state of the system at that time and on the time instant *t*, that is, that $A_i(t)$ is a deterministic function of *X*(*t*) and of *t*, the network formation process of the two time-varying layers at time *t* is a function of *X*(*t*) and of *t*. Hence, the stochastic process *X*(*t*) is ultimately a Markov chain on the state space $\{S,E,I_D,I_U,R,V\}^n$, whose state transitions are governed by Eqs. (4) and (7). Furthermore, if $A_i(t)$ depends on *t* only through *X*(*t*), then the Markov chain is time-invariant (Levin et al. 2006). The latter is the case in which home-isolation policies are feedback of the state (for instance, if detected individuals, their family members, and/or their classmates are home-isolated), but no time-dependent policy is enacted (for instance, school attendance on alternating days or weeks). The Markovianity of the process *X*(*t*) allows performing fast simulations of the systems, to shed light on the role of children and schools in the transmission of the disease, and to investigate the effectiveness of different home-isolation and vaccination strategies, as illustrated in “Results” section.

## Model calibration to COVID-19 in France and simulation setting

We demonstrate the potentialities of our modeling framework by studying some scenarios inspired by the spring 2021 vaccination campaign against COVID-19 in France. In this case study, we consider a daily temporal granularity, that is, each time-step coincides with a day. Hence, before presenting the findings of our simulation studies, we provide some details on the model calibration, based on demographic and epidemiological data, and on the setting we have designed to perform the simulations.

### Population and network

We generate a network composed of $k=50,000$ families. Families are generated according to France census data from the French National Institute of Statistics and Economic Studies (*Institut national de la statistique et des études économiques*) (https://www.insee.fr/fr/statistiques/4277630?sommaire=4318291&fbclid=IwAR3z-EUWTcRXgeE5VK-XE3Mkk6SugqJXZG1ox4r0qi7tRo220DpvLErRKvY). Specifically, census data report that 35.8% of the families are formed by a single member, 6% by single adults with one or more children, 21% by a couple with one or more children, and 36.7% by two or more adults without children. Accordingly, we determine that 13,500 families have children (27%): 3000 of them include a single adult, while the others 13,500 have two adults. Then, to each of these 13,500 families, we assign a variable number of children $n_s$, generated from a zero-truncated Poisson random variable (r.v.) with an expected value equal to $\langle n_s \rangle = 1.79$, each one independent of the others. Such a variable is calibrated on the average number of school children for families in France (https://www.insee.fr/fr/statistiques/4277630?sommaire=4318291&fbclid=IwAR3z-EUWTcRXgeE5VK-XE3Mkk6SugqJXZG1ox4r0qi7tRo220DpvLErRKvY). Of the remaining 36,500 families, 17,900 (36.7%) are formed by a single adult. The remaining 18,350 families are formed by a variable number of children $n_a$, generated from a one-truncated Poisson random variable (r.v.) with an expected value equal to $\langle n_a \rangle = 2.41$, each one independent of the others. This variable is calibrated to fit the average family size in France, according to France census data (https://www.insee.fr/fr/statistiques/4277630?sommaire=4318291&fbclid=IwAR3z-EUWTcRXgeE5VK-XE3Mkk6SugqJXZG1ox4r0qi7tRo220DpvLErRKvY). Note that the total number of individuals in the network *n* is a r.v. with an expected value equal to $\langle n \rangle =110,000$, being equal to 85,835 adults and the 24,165 children, on average.

The children are randomly associated with their classes. Specifically, the number of children in each class is set to be $n_c=24$, consistent with the average class size in French schools (https://www.education.gouv.fr/les-chiffres-cles-du-systeme-educatif-6515). We randomly partition the classes in buildings with 20 classes each. The school building layer is generated according to the algorithm described in the previous section. Specifically, the activity of $i\in {\mathcal {C}}$ is a power-law distributed r.v. with lower-barrier at $a_{\min }=0.1$, upper-barrier at $a_{\max }=1$, and exponent equal to $\alpha =-2.09$, as in Aiello et al. (2001) (additional simulations to demonstrate the robustness of our findings with respect to different choices of the parameter of the power-law are reported in the “Appendix”, Fig. 8). The number of connections initiated by each active individual is set to be equal to $m_c=7$. Such a variable is calibrated on the SocioPatterns primary school dataset (Stehlé et al. 2011), by considering the average daily number of contacts between students in different classes that last for a sufficiently long time. Specifically, consistent with the European Union guidelines on the definition of contacts at risk (https://ec.europa.eu/health/ehealth/covid-19_en), we have only considered contacts that last cumulatively at least 15 min. Fourth, while we have explored some potentialities of our modeling framework, a fundamental and complete analysis of the proposed model is still missing. Despite its complexity may hinder the analytical derivation of theoretical results, we believe that its amenability to perform fast simulations poses the basis for future numerical studies on the general properties of the model, including the numerical computation of the epidemic threshold.

The contact layer is generated according to a discrete-time ADN (Perra et al. 2012). Specifically, the activity of $i\in {\mathcal {A}}$ is a power-law distributed r.v. with lower-barrier at $a_{\min }=0.1$, upper-barrier at $a_{\max }=1$, and exponent equal to $\alpha =-2.09$, as in Aiello et al. (2001). The number of connections initiated by each active individual $m_a$ is computed as follows. Based on empirical observations, the authors in Mossong et al. (2008) identify that active adults have on average 18 daily interactions (including those within the family, and those initiated by other individuals). Hence, we set the value of $m_a$ such that the expected degree of active adults matches with this estimation, rounded to the closest integer. Specifically, we enforce

8 $$\begin{aligned} 18=(1+\langle a\rangle )m_a+\langle k_{f} \rangle \implies m_a = \text {round}\left( \frac{18 - \langle k_{f} \rangle _a }{1+\langle a \rangle }\right) =13\,, \end{aligned}$$

where

9 $$\begin{aligned} \langle a \rangle = \frac{\int _{a_{\min }}^1 x^{1+\alpha } dx}{\int _{a_{\min }}^1 x^{\alpha } dx}= \frac{\int _{0.1}^1 x^{-1.09} dx}{\int _{0.1}^1 x^{-2.09} dx}=0.247 \end{aligned}$$

is the average activity, and

10 $$\begin{aligned} \langle k_f \rangle =1.56 \end{aligned}$$

is the average number of links of adults on the family layer, computed from the distribution of adults in families described in the above.

We can compute the average degree for children an adults in the absence of home-isolation as the sum of the degrees on the four layers, that is,

11 $$\begin{aligned} \langle k \rangle _a = \left( \langle k_f \rangle _a+2 \langle a \rangle m_a \right) =7.98 \end{aligned}$$

and

12 $$\begin{aligned} \langle k \rangle _c = \left( \langle k_f \rangle _c + \frac{5}{7}\left( \langle n_c\rangle -1 +2 \langle a \rangle m_c \right) \right) = 21.9, \end{aligned}$$

respectively, where the term 5/7 accounts for school closure in the weekend, and $\langle k_f \rangle =3$ is the average number of links of children on the family layer computed from the distribution of adults in families described in the above. Finally, the average degree of an individual is obtained as their weighted average, that is,

13 $$\begin{aligned} \langle k \rangle = \frac{85,835}{110,000}\langle k \rangle _a+\frac{24,165}{110,000} \langle k \rangle _c= 15. \end{aligned}$$

### COVID-19 epidemic parameters

The epidemic parameters $\lambda$, $\nu$, and $\mu$ for COVID-19 are set from epidemiological data as follows. Reliable estimations of the average latency period $\tau _E=6.4$ days and of the average period of communicability $\tau _I=5$ days are available Prem (2020). From these data, similar to Prem (2020), we obtain

14a $$\nu =1-\exp \left( -\frac{1}{\tau _E}\right) =0.1447,$$

14b $$\mu =1-\exp \left( -\frac{1}{\tau_I}\right) =0.1813.$$

The per-contact infection probabilities $\lambda _c$ and $\lambda _a$ are estimated from the basic reproduction number $R_0$, which is the average number of secondary infections generated by an infectious individual, assuming that the rest of the population is susceptible and in the absence of NPIs. Note that estimating the per-contact infection probabilities from the basic reproduction number (and not from the effective reproduction number) allows us for avoiding the possible confounding due to the effect of NPIs on the contagion process. Hence, since $\tau _I$ is the average time that an individual is infectious, we write

15 $$\begin{aligned} R_0=\left( \frac{{\tilde{n}}}{n}\lambda _c \langle k \rangle _{c}+\frac{n-{\tilde{n}}}{n}\lambda _a \langle k \rangle _{a}\right) \tau _I. \end{aligned}$$

In this paper, we consider three possible scenarios, making different assumptions on the contagiousness of children and adults, and considering either the original strain and the Alpha variant (B 1.1.7), which quickly became dominant in Europe in spring 2021, during the vaccination campaign.

**Scenario I** (**original strain, uniform contagion probability**) In the first scenario, we consider the original COVID-19 strain, and we assume that children and adults have the same per-contact infection probability, that is $\lambda _a=\lambda _c={\bar{\lambda }}$. According to reliable results on the estimation of the basic reproduction number (Zhang et al. 2020), we set $R_0=2.28$. In this scenario, by inverting (15) we obtain ${\bar{\lambda }}=0.040$.

**Scenario II** (**original strain, different contagion probability**) In the second scenario, we consider the original COVID-19 strain ($R_0=2.28$), and we assume that children are less contagious than adults. Empirical studies suggest that children may have a transmissibility reduced by 37%, with the extreme value of the 95% confidence interval (CI) at 63% Dattner et al. 2021; (15) yields $\lambda _a=0.047$ and $\lambda _c=0.029$. The corresponding 95% CI are $\lambda _a\in [0.040,0.053]$ and $\lambda _c\in [0.020,0.040]$.

**Scenario III** (**Alpha variant**) In the third scenario, we consider the Alpha variant (B 1.1.7), which is estimated to be more infectious than the original one (Davies et al. 2021), with a increased transmissibility increased by 43–90% (95% CI). We assume that children and adults have the same per-contact infection probability, that is $\lambda _a=\lambda _c={\bar{\lambda }}_{\alpha }$. In this scenario, we obtain ${\bar{\lambda }}_{\alpha }\in [0.057,0.076]$, with an average of ${\bar{\lambda }}_{\alpha }=0.067$.

The detection rates $q_a$ and $q_c$ are control parameters that reflect the effort and the effectiveness of testing practices and they are lower-bounded by the symptomatic rates $q_a\ge 0.25$ and $q_c\ge 0.12$, as suggested in (https://www.edgehealth.co.uk/post/as-many-as-1-in-5-people-in-england-have-had-the-covid-19-disease) and Han et al. (2021), respectively.

Note that, in our disease progression, we assume that individuals become immune after recovery. Such an assumption is consistent with recent clinical analysis of COVID-19, which suggest that immunity lasts on average at least 6–8 months (i.e., the same duration of the time-horizon of our simulations) (Gudbjartsson 2020; Dan 2021).

### Home-isolation policies

Our work considers the implementation of different home-isolation policies. Differently from vaccinations, which are set at the beginning of each simulation, isolation policies are dynamical. Common to all policies we have that individuals that are detected ($I_D$), are always home-isolated, that is,

16 $$\begin{aligned} X_i(t)=I_D\implies A_i(t)=0. \end{aligned}$$

Besides this basic rule, further policies may be enacted to enforce home-isolation of non-detected individuals that may be (potentially) infected. Specifically, we consider the two policies described in the following.

**Policy A** (**family-isolation policy**) Under this policy, when an individual is detected, then all their family members are home-isolated, that is

17 $$\begin{aligned} X_i(t)=I_D\implies A_j(t)=0,\quad \forall j\in \phi (i). \end{aligned}$$

**Policy B** (**class-isolation policy**) Under this policy, when an individual is detected, then all their schoolmates are home-isolated, that is

18 $$\begin{aligned} X_i(t)=I_D\implies A_j(t)=0,\quad \forall j\in \psi (i). \end{aligned}$$

In all our simulations, we assume that the family-isolation rule is enacted, while in some of the simulations, we will also enforce the class-isolation policy. We will explicitly report when both policies are implemented.

### Vaccination strategies and initialization

In all our simulations, we initialize the system by setting a fraction $V\in [0,1]$ of the population in the vaccinated (*V*) health state. Such a fraction may vary across the simulation settings, as detailed in the next section, when describing the results. Fixed the fraction of vaccinated individuals, the following three different vaccination strategies are considered and compared.

**Vaccination strategy I** (**uniform adult vaccination**) A fraction *V* of the population chosen uniformly at random among the adults is vaccinated.

**Vaccination strategy II** (**uniform vaccination**) In this strategy, the fraction *V* is chosen uniformly at random among the entire population, including children. Even though this strategy is not realistically viable in the current stage of the vaccination campaign (https://www.nytimes.com/2021/02/12/health/covid-vaccines-children.htm), its implementation in our simulations allows us to better understand the impact of children in the spreading of COVID-19.

**Vaccination strategy III** (**targeted vaccination**) Similar to the uniform adult vaccination, a fraction *V* of the population is chosen for vaccination. However, the individuals are not selected uniformly at random, but vaccinated in decreasing order of size of the corresponding families.

Note that, for different vaccination policies, the population eligible for vaccination is different. In particular, while for I and III the eligible population coincides with all the adults, for II the entire population is eligible to be vaccinated.

More realistic vaccination strategies can be modeled in a dynamic fashion, by starting the vaccination campaign in a specific date (possibly after the beginning of the simulations) and adding a transition mechanism from the susceptible state to the vaccinated one (possibly time-varying to model the increased vaccination capacity of the healthcare system and the growing supplies), and by including further features such as vaccine hesitancy and other orders of prioritization. These features could be easily incorporated within our modeling framework, following implementations similar to the one proposed in the recent literature (Grauer et al. 2020; Bubar et al. 2021; Truszkowska et al. 2021; Foy et al. 2021; Parino et al. 2021). Here, however, we opt to model vaccination in a simplified and “static” fashion. Such a choice allows us to reduce the impact of possible confounding variables added by the dynamic vaccination process, and thus focus our analysis on the role of schools and children on the disease spreading, given that a certain stage of the vaccination rollout has been reached. For the sake of discussing the flexibility of our modeling framework and the robustness of our findings, simulation results with simple dynamic vaccination policies are reported in the “Appendix”.

The rest of the population, that is, those not vaccinated, are initialized by randomly assigning 1% of the population to the exposed health state (*E*), compatible with estimations on the number of active COVID-19 cases in France (including undetected individuals) as of May 2021 (https://dashboard.covid19.data.gouv.fr), while all the others are susceptible (*S*). In the “Appendix”, we report some additional simulations to show that the choice of the initial condition has a negligible impact on the epidemic process. The parameters common to all the simulations are reported in Table 2.

**Table 2:** Value of the parameters used in the simulations. The last three parameters, namely $q_a$, $q_c$, and *V*, vary across the simulations and their values are explicitly reported when presenting the results

	Meaning	Value
*k*	Number of families	50,000
$n_s$	Children in each family with children	Zero-truncated Poisson r.v., mean 1.79
$n_a$	Adults in each family with no children	One-truncated Poisson r.v., mean 2.41
$n_c$	children in each class	24
$a_i$	Activity of node *i*	[0.1, 1], power law r.v., exponent $-2.09$
$m_a$	Interactions of active adults	13
$m_c$	Interactions of active children	7
${\bar{\lambda }}$	Per-contact infection probability (S I)	0.040
$\lambda _a$	Adult per-contact infection probability (S II)	0.047 (95% CI [0.040,0.053])
$\lambda _c$	Children per-contact infection probability (S II)	0.029 (95% CI [0.020, 0.040])
${\bar{\lambda }}_{\alpha }$	Per-contact infection probability (S III)	0.067 (95% CI [0.057,0.076])
$\nu$	Probability of becoming infectious	0.1447
$\mu$	Recovery probability	0.1813
$q_a$	Adults detection rate	–
$q_c$	Children detection rate	–
*V*	Population vaccinated	–

## Results

In this section, we utilize the model calibrated to COVID-19 to investigate several scenarios. Specifically, we will devote the first set of experiments toward shedding light on the role of children in the spreading of COVID-19. Then, the second set of simulations is performed to explore the effectiveness of increasing the testing of adults and children, showing that massive testing campaigns among children are necessary to effectively reduce the contagions. Then, we assess the performance of the *class-isolation* policy presented in subsection Home-isolation policies, demonstrating that it seems an effective strategy to flatten the epidemic curve without the need for massive (and often unfeasible) testing campaigns. Finally, we compare the uniform adult vaccination strategy with the prioritization of large families proposed in subsection Vaccination strategies and initialization, showing that the proposed strategy might be a viable strategy to reach herd immunity faster.

### Key role of children in the spreading of COVID-19

In the first set of experiments, we investigate the role of children in the spreading of COVID-19. To remove any other confounding elements, we set the detection rates at their minimal values, coinciding with the symptomatic rates, that is, $q_a = 0.25$ and $q_c=0.12$, and we consider the simplest home-isolation policy A, described in subsection Home-isolation policies, in which only family members of detected individuals are enforced to home-isolate themselves. To highlight the role of children in the spreading process, we report the fractions of infections among children and adults separately.

**Fig. 4:**
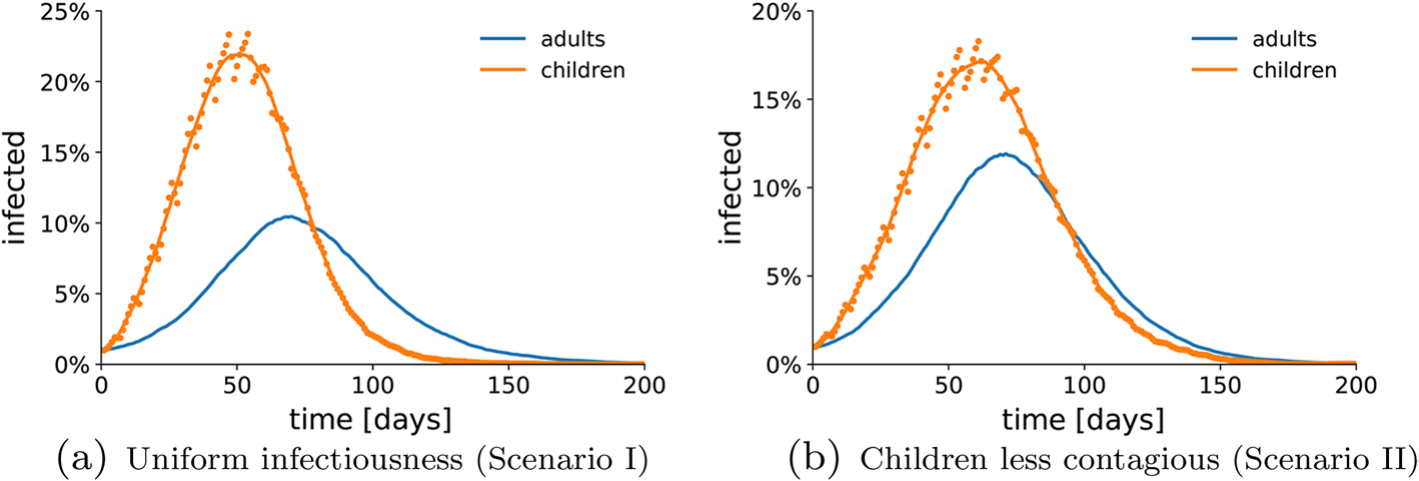
Role of children in the spreading of COVID-19. We show the temporal evolution of the fraction of infections among the adults (blue dots) and children (orange dots) in a representative simulation, in the absence of vaccination $V=0$. The solid curves illustrate the 7-day moving average of the two quantities. In (**a**), we consider Scenario I (original strain, uniform contagion probability), in which all the individuals have the same per-contact infection probability; in (**b**), Scenario II (original strain, different contagion probability), in which children have a decreased per-contact infection probability. The parameters used in the simulations are listed in Table 2, $q_a=0.25$, and $q_c=0.12$

Figure 4 reports the temporal evolution of the number of infections among the children and the adults in the absence of vaccination ($V=0$), both under the assumption that children and adults have the same per-contact infection probability (Fig. 4a), and assuming that children have a lower infection probability (Fig. 4a). Both simulations show that the infections among children grow faster, while the infections among adults seem to follow the children’s wave. This observation intuitively suggests that control of the children spreading might be crucial toward successfully flattening the epidemic curve.

**Fig. 5:**
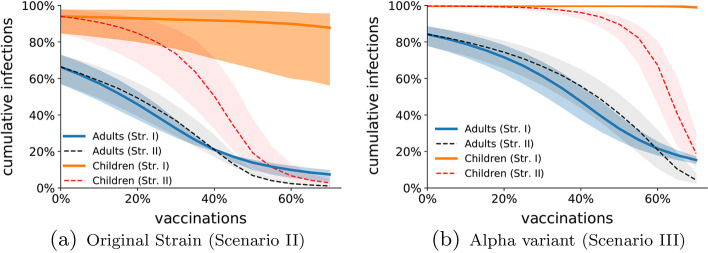
Effect of children vaccination. We show the Monte Carlo estimation (over 100 independent simulations) of the cumulative fraction of infections among non-vaccinated adults (blue) and children (orange) at the end of the pandemic outbreak, as a function of the fraction of vaccinated population *V*. The solid curves refer to Vaccination strategy I, in which only adults are eligible for vaccination; the dashed curve refers to Vaccination strategy II, in which vaccine shots are randomly assigned to the entire population. In (**a**), we consider the original strain; the vertical bands represent the confidence interval with respect to the decreased infectiousness of children. In (**b**), we consider the Alpha variant; the vertical bands represent the confidence interval with respect to the increased infectiousness of the Alpha variant. The parameters used in the simulations are listed in Table 2, $q_a=0.25$, and $q_c=0.12$

To support our intuition, we design a set of experiments to investigate the difference between two vaccination strategies, and we perform a set of Monte Carlo simulations of the epidemic process for different fractions of the vaccinated population—spanning from no vaccinations to vaccinating 70% of the population. Specifically, we compare strategy I, in which only adults are vaccinated, and strategy II, in which all the population is eligible for vaccination, for both the original strain (Scenario II, in Fig. 5a) and the Alpha variant (Scenario III, in Fig. 5b).

The results of our simulations, illustrated in Fig. 5, show some non-trivial behaviors. Specifically, our simulations suggest that Strategy I (vaccinating only adults, solid curves in the figures) has a weak overall impact on the epidemics, as the cumulative number of infections among children is marginally reduced, even when the majority of the adult population is vaccinated. This suggests that it might be difficult to reach herd immunity, without vaccinating children (or implementing specific policies to reduce contagions in schools, as we shall discuss in the following). On the contrary, Strategy II (vaccinating all the individuals, dashed curves in the figures) seems to drastically impact the course of the outbreak, as it allows to reduce the cumulative number of infections in both groups, leading to herd immunity when about 60% and 70% of the population is vaccinated, for the original strain and the UK one, respectively. Interestingly, while in the early stages of the vaccination campaign Strategy I seems to be beneficial in reducing the number of cumulative infections at least among adults, when the vaccination rollout reaches a critical fraction (close to 40% and 60% for the two strains, respectively), Strategy II seems to outperform Strategy I not only among children, but also among adults (even though Strategy II entails fewer vaccinations among adults than Strategy I). We believe that this phenomenon is due to the increasing herd immunity gained by children corresponding to the sharp decrease in the number of cumulative infections among children that can be observed in Fig. 5a when 30% of the population is vaccinated (50% for the Alpha variant in Fig. 5b). Such a sharp decay strongly impacts the contagions within the family.

Unfortunately, the above-mentioned strategy, although attractive, could be of difficult application in the case of the COVID-19 pandemic, being children excluded from the trials of the first vaccines https://www.nytimes.com/2021/02/12/health/covid-vaccines-children.htm). We also would like to stress that similar issues will be likely faced also with future novel viruses, for which new drugs and vaccines will be first tested on adults. Therefore, in the next sections, we will test alternative strategies to mitigate the disease, which do not entail the vaccination of children.

### Flattening the curve through testing and home-isolation policies

**Fig. 6:**
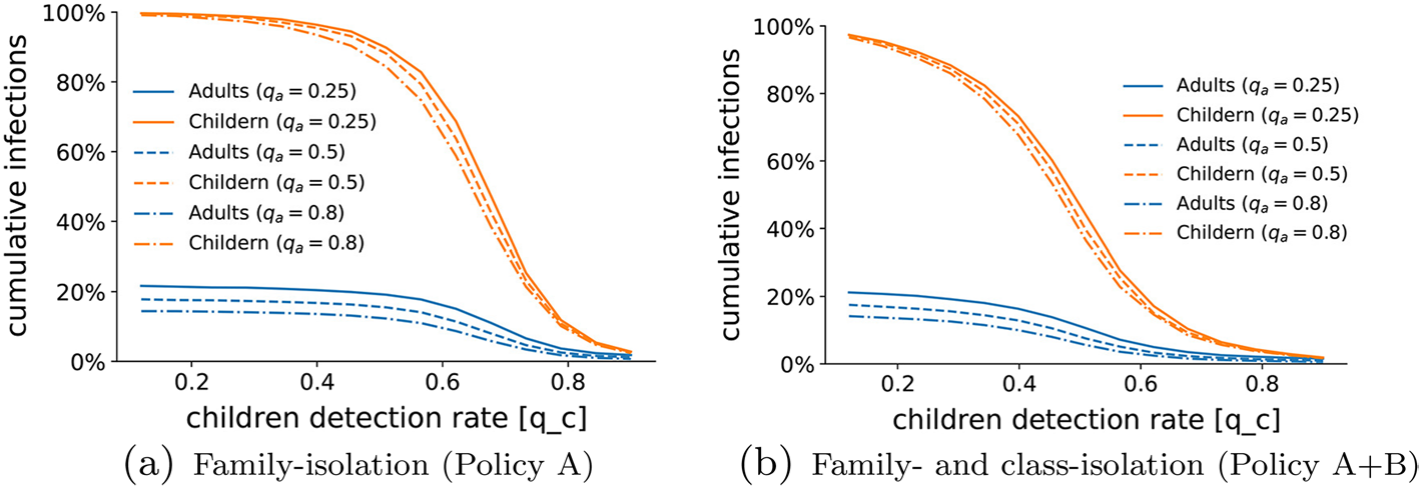
Effect of testing and different home-isolation policies. We show the Monte Carlo estimation (over 100 independent simulations) of the cumulative fraction of infections among adults (blue) and children (orange) at the end of the pandemic outbreak as a function of the children detection rate $q_c$ and for three different values of adult detection rate, representative of low testing $q_a=0.25$ (solid), moderate testing $q_a=0.5$ (dashed), and massive testing $q_a=0.8$ (dash-dotted). In (**a**), we utilize the family-isolation policy (Policy A). In (**b**), we consider a scenario in which also the class-isolation policy (Policy B) is present. The comparison of the two panels shows that the application of both policies (dashed) sensibly outperforms utilizing only A (solid). The parameters used in the simulations are listed in Table 2 and $V=0.6$. All the simulations are done in Scenario III (Alpha variant), utilizing the average infectiousness reported in Table 2

In the second set of experiments, we investigate the possibility of flattening the epidemic curve by means of increasing the testing capacity and implementing effective home-isolation policies. In our simulation study, we thus fix the fraction of the vaccinated population at 60% of the entire population ($V=0.6$), selected uniformly at random among the adults according to vaccination strategy I and we consider the Alpha variant (Scenario III). Then, we first explore different testing strategies by estimating the cumulative number of infections for different values of $q_a\in \{0.25,0.5,0.8\}$ and $q_c\in [0.12,1]$. The results of our analysis, illustrated in Fig. 6a, suggest that, even when a non-negligible fraction of the population is already vaccinated, massive outbreaks are still possible. Moreover, we observe that the ability to detect most of the infections among adults is not sufficient to flatten the epidemic curve, as only a mild decrease in the number of cases is observed. On the contrary, massive screening campaigns among children seem to be effective in reducing the cumulative number of infections, not only among children but within the entire population. However, according to this model, effective screening policies should be able to detect at least 80% of the infections among children, which may be realistically unfeasible or extremely costly, considering the large number of tests that such a practice would require to process (Pullano et al. 2020).

To address this issue, we utilize our model to investigate whether targeted home-isolation policies for children can be enacted to reduce the testing effort needed to flatten the epidemic curve. Specifically, we consider combining the family-isolation policy A with the school-isolation policy B, described in subsection Home-isolation policies, in which not only the family members of detected individuals have to home-isolate, but also all the classmates of children that are detected are home-isolated until their infected mate has recovered. The results are illustrated in Fig. 6b. Predictably, this strategy has a moderate impact for low values of $q_c$, since only a small fraction of the infected children could be detected, and thus few classes are home-isolated. As the children detection rate $q_c$ increases, the fraction of infections (among children and adults) abruptly drops down, and a detection rate of 60% seems to be sufficient to guarantee successful mitigation of the outbreak. This result compares favorably to our previous findings, since it seems that disease mitigation can be achieved with more realistic testing practices. Note that, even in this scenario, increasing the adult detection rate has a minor impact on the outcome of the outbreak.

### Targeted vaccination campaign

Finally, we hypothesize that the latter strategy can be further improved by prioritizing the vaccination of families with a large number of sons. To test this hypothesis, we compare the outcome of the uniform adult vaccination strategy I, with the targeted vaccination strategy III, in which vaccinations are performed on adults, prioritizing members of large families. It is worth stressing that this approach is way more simple than prioritizing vaccination of the most active individuals based on their social activity pattern (as proposed in Liu et al. 2014), being the size of each family a non-ambiguous number, easily accessible for public health authorities and regulators. All these simulations are performed for the Alpha variant (Scenario III).

**Fig. 7:**
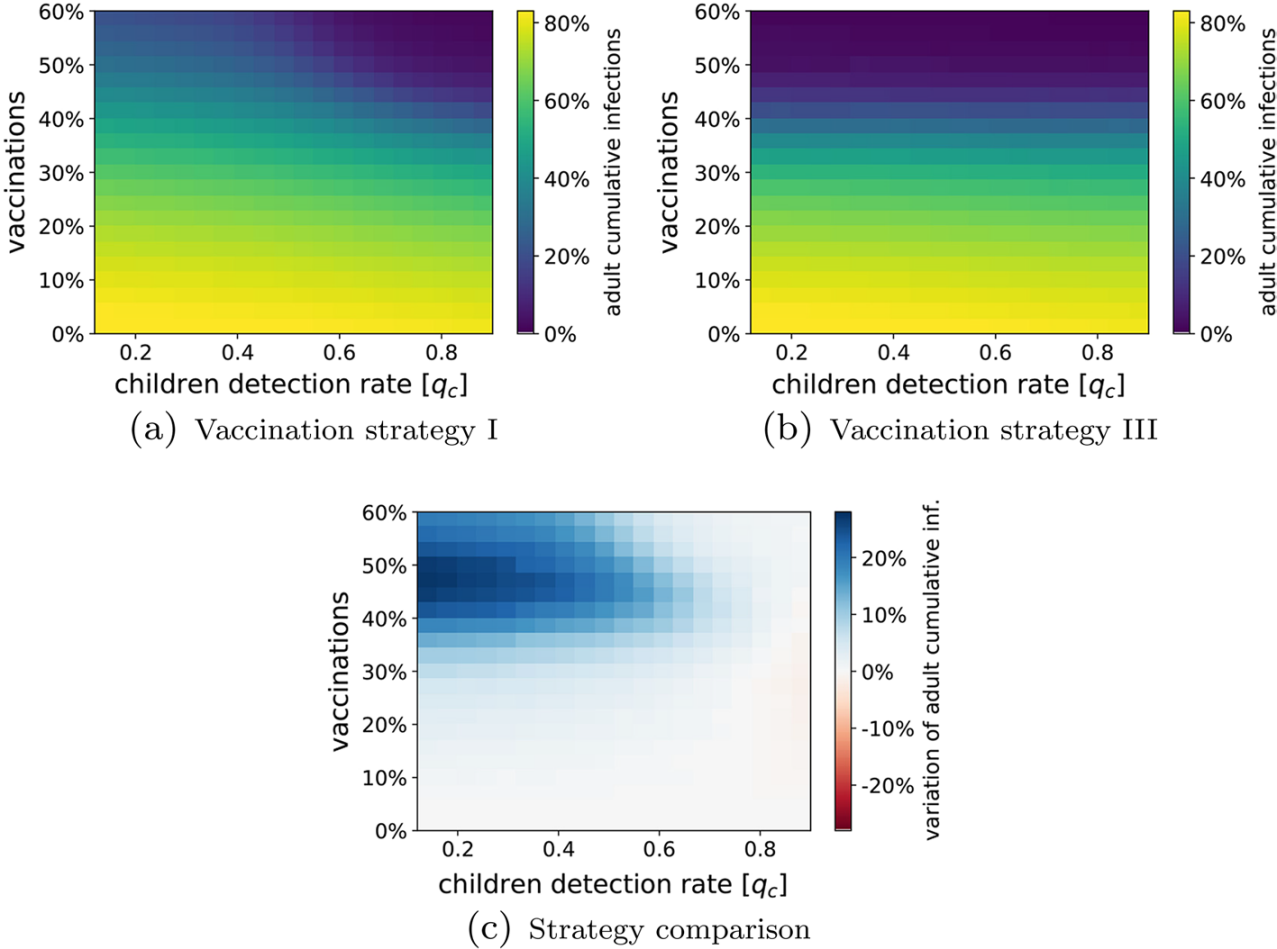
Comparison between different vaccination strategies. In (**a**) and (**b**), we show the Monte Carlo estimation (over 100 independent simulations) of the cumulative number of infections among adults at the end of the epidemic outbreak, for different values of the fraction of eligible individual vaccinated (*V*) and different children detection rate ($q_c$). In (**a**), we adopt vaccination strategy I, in which the vaccinated individuals are selected uniformly at random among the adult population. In (**b**), we adopt vaccination strategy III, in which the vaccination of adults belonging to large families is prioritized. In (**c**), the two strategies are compared, showing the variation in the cumulative numbers of infections between the two strategies. The parameters used in the simulations are listed in Table 2 and $q_a=0.25$. All the simulations are done in Scenario III (Alpha variant), with the average infectiousness reported in Table 2

**Fig. 8:**
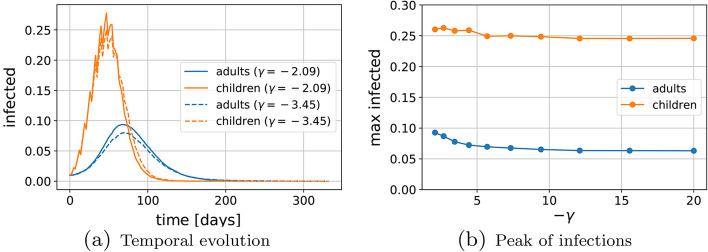
Role of the negative exponent of the power-law distribution in the ADNs. In (**a**), we show the temporal evolution of the number of infected children and adults (averaged over 10 runs), for two different choices of the power-law exponent. In (**b**), we show the peak of the epidemic prevalence (estimated over 100 runs), for increasing values of the negative exponent of the power law. All simulations are performed in the absence of vaccination $V=0$ for Scenario I (original strain). The parameters used in the simulations are listed in Table 2, $q_a=0.25$, and $q_c=0.12$

In Fig. 7b, we show a heat-map of the cumulative number of infections among adults at the end of the outbreak under the two vaccination strategies, for different values of the children detection rate $q_c$ and of the fraction of population vaccinated *V*. Under Vaccination strategy I (uniform adult vaccination), the figure clearly depicts a trade-off between children testing and vaccinations. In plain words, when a small fraction of the adult population is vaccinated, a massive testing campaign on children is necessary to control the spreading; then, when the vaccine has reached a sufficiently large portion of the adult population, the effort placed in children testing can be reduced. Under Vaccination strategy III (prioritization of large families), instead, the children detection rate seems to play a less important role.

In fact, when comparing the two vaccination strategies, we observe that, in many scenarios, strategy III (namely, the targeted vaccination) outperforms strategy I (the uniform one), as can be observed in Fig. 7. In particular, while in the very early stages of the vaccination campaign strategy I may provide a very small benefit (in particular in the presence of massive testing campaigns), strategy III becomes more efficient when about 30% of the population is vaccinated, and becomes more and more advantageous as the number of vaccinated individuals increases, in particular for smaller values of children detection rate. For instance, when 40% of the population is vaccinated, the targeted vaccination strategy reduces the infections up to 20%, when no testing campaigns are implemented. Interestingly, the vaccination strategy that prioritizes large families seems to yield herd immunity with 50% of the population vaccinated, whereas a similar result with the uniform vaccination strategy requires a much higher number of vaccinations, or a massive (and unrealistic) children testing ($q_C>0.7$).

## Discussion and conclusions

In this paper, we have proposed a stochastic multi-layer network model for the spreading of epidemic diseases. The model is specifically oriented toward studying the role of children and schools in the spread of airborne diseases. To this aim, the proposed model incorporates four layers of social contacts to account for interactions between family members, within classes, casual interactions between children in their school building, and between adults, respectively. The network model is then combined with a tunable disease progression model that accounts for a limited detectability of infectious individuals, inherent differences between adults and children, the possible implementation of home-isolation policies, and vaccination. The proposed model is general and flexible, enabling its use to perform different analyses and investigate what-if scenarios toward understanding the role of schools in the spreading of epidemic diseases, and assessing different vaccination strategies, testing practices, and home-isolation policies.

We have demonstrated the potentiality of our modeling framework by analyzing a case study, inspired by and calibrated on the spring 2021 COVID-19 pandemic and vaccination campaign in France (https://www.insee.fr/fr/statistiques/4277630?sommaire=4318291&fbclid=IwAR3z-EUWTcRXgeE5VK-XE3Mkk6SugqJXZG1ox4r0qi7tRo220DpvLErRKvY, https://dashboard.covid19.data.gouv.fr, https://www.education.gouv.fr/les-chiffres-cles-du-systeme-educatif-6515). During this vaccination campaign, NPIs are still implemented (reducing thus the number of contagions in other locations), while schools are open for in-person education. Echoing the concerns of several researchers (Gurdasani et al. 2021; Hyde 2020), we hypothesize that schools may play a key role in the spreading of the disease. Under this hypothesis, implementing intervention policies oriented to prevent contagions in schools—such as targeted home-isolation strategies and testing campaigns—is crucial to flatten the epidemic curve. We thus utilize our modeling framework to test such a hypothesis, by means of a campaign of Monte Carlo numerical simulations.

Our findings have confirmed our hypothesis. Specifically, they have provided novel insights into the role of schools on the spreading of epidemics and into the effectiveness of different testing practices and home-isolation policies. First, our model, has allowed us to disentangle the role of children and adults in the spreading process. Specifically, we have shown that during the phase considered in the case study, contagions in schools are key drivers of the epidemics and intervention policies aiming at reducing the transmissions between children are thus crucial toward mitigating the spread. Considered the impossibility of vaccinating children in the early stages of a vaccination campaign (https://www.nytimes.com/2021/02/12/health/covid-vaccines-children.htm), the only way to control contagions in schools is by means of testing campaigns and implementing targeted temporary online education. In this vein, our second main result suggested that a massive testing campaign of children may be effective in flattening the epidemic curve, in particular, when testing is combined with the implementation of temporary online education for the classes in which infectious children are detected. With the implementation of such a home-isolation practice, we have shown that a reasonable testing effort (able to detect about 60% of the infected children) is sufficient to keep the pandemic under control (even considering the more infectious Alpha variant of COVID-19). Finally, we have tested the effectiveness of a targeted vaccination policy. In the proposed strategy, the vaccination of adults in large families is prioritized. The goal of such a policy is to reduce the probability of generating epidemic clusters in large families, whose many children go to different classes and could potentially infect a large number of individuals. Such a strategy may possibly avoid those super-spreading events that are typical of the inception of outbreaks of airborne diseases, including COVID-19 (Wong and Collins 2020). The results of our simulations have confirmed our intuition that the proposed vaccination strategy might be beneficial to reduce the number of infections, favoring the faster reaching of herd immunity.

When evaluating the outcome of our study, one should carefully acknowledge its limitations. First, the model relies on the simplifying assumption that all interactions yield the same transmission probability (with a possible differentiation for adults and children), irrespective of their duration, intensity, and location in which they occur. However, the duration and intensity of social contacts may impact the transmission probability, while interactions in different locations may be associated with different risks. We believe that our framework can be extended by assigning weights to the links (i.e., by considering a weighted network) to account for heterogeneity in the duration and intensity of interactions, and by using location-dependent infection probabilities. Second, in our model, we assume that the country is in partial lockdown, that is, most of the adults work from home or in safe environments and most of the non-essential activities are canceled (e.g., sports and cultural activities, large gatherings). Even though, at the beginning of the 2021 COVID-19 vaccination campaign many countries are undergoing a (partial or total) lockdown due to the ongoing second and third epidemic waves, addressing similar issues for future questions (e.g., a potential booster dose vaccination campaign) may require to consider scenarios with the relaxation of the current NPIs. Thanks to the flexibility of our model, further sources of social interactions—and, thus, of potential contagions—can be incorporated within our modeling framework by including additional layers in the multi-layer network to account for interactions in leisure and non-essential locations, similar to Truszkowska et al. (2021). This, allows to capture, for instance, social interactions in work-places, in sport and cultural centers, and between groups of friends. Third, in this paper, we consider simplified vaccination strategies, which do not take into account important factors such as prioritization of high-risk groups and the presence of vaccine hesitancy. The inclusion of these factors within our modeling framework would be a necessary step toward performing further studies and designing realistic vaccination campaigns. Such inclusion may be implemented by explicitly incorporating a vaccination mechanism that makes individuals dynamically transition from the susceptible state to the vaccinated one. Fourth, the multi-layer network structure is tailored to capture the French socio-demographic and educational system. Hence, some adjustments may be required to apply it to different social and educational systems, such as the US one, in which colleges play a key role.

To conclude, in this work we have focused on the role of schools in the spreading of infectious diseases, highlighting how some intervention policies and vaccination strategies can be beneficial for mitigating the epidemics. Our work can thus be relevant to help inform public health authorities in their decisions. We have also discussed the main limitation of our work, discussing how our formalism can be easily generalized to incorporate further real-world features, without any fundamental change in its main mechanisms. Such a flexibility would allow the scientific community to utilize our modeling framework and adapt it to the analysis of future issues related to the control of COVID-19 and to increase preparedness for future epidemic outbreaks.

## Figures and Tables

**Fig. 9:**
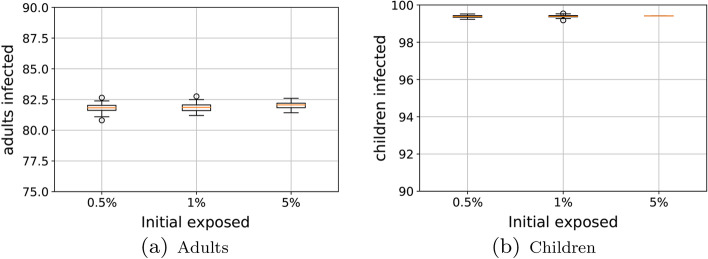
Role of the initial condition. We show the cumulative number of infections (over 100 independent runs) among (**a**) adults and (**b**) children, for three different initial conditions. Our findings suggest no statistically significant difference between the outcomes of the simulations. All simulations are performed in the absence of vaccination $V=0$ for Scenario I (original strain). The parameters used in the simulations are listed in Table 2, $q_a=0.25$, and $q_c=0.12$

**Fig. 10:**
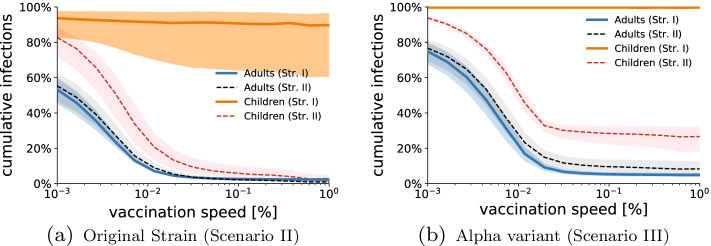
Effect of vaccination speed. We show the Monte Carlo estimation (over 100 independent simulations) of the cumulative fraction of infections among all adults (blue) and children (orange) at the end of the pandemic outbreak, as a function of the vaccination speed in percentage of the total population daily vaccinated, the vaccination is stopped when $V=60\%$. The solid curves refer to Vaccination strategy I, in which only adults are eligible for vaccination; the dashed curve refers to Vaccination strategy II, in which vaccine shots are randomly assigned to the entire population. In (**a**), we consider the original strain; the vertical bands represent the confidence interval with respect to the decreased infectiousness of children. In (**b**), we consider the Alpha variant; the vertical bands represent the confidence interval with respect to the increased infectiousness of the Alpha variant. The parameters used in the simulations are listed in Table 2, $q_a=0.25$, and $q_c=0.12$

**Fig. 11:**
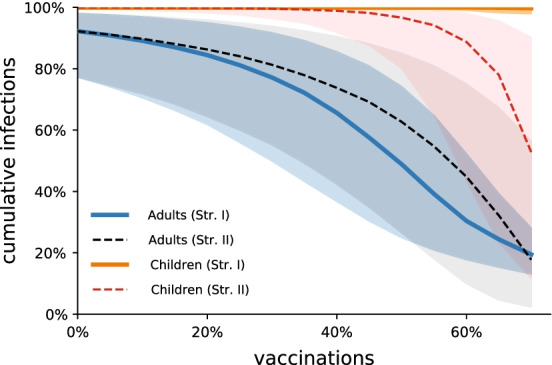
Scenario of the Delta variant. We show the Monte Carlo estimation (over 100 independent simulations) of the cumulative fraction of infections among non-vaccinated adults (blue) and children (orange) at the end of the pandemic outbreak, as a function of the fraction of vaccinated population *V*. The solid curves refer to Vaccination strategy I, in which only adults are eligible for vaccination; the dashed curve refers to Vaccination strategy II, in which vaccine shots are randomly assigned to the entire population. The vertical bands represent the confidence interval with respect to the increased infectiousness of the Delta variant (parameters reported in the “Appendix”). The other parameters used in the simulations are listed in Table 2, $q_a=0.25$, and $q_c=0.12$

## Data Availability

The simulation code is available at https://gitlab-research.centralesupelec.fr/2019bongiornc/schoolcovidsim.

## Appendix

In this appendix, we provide some additional simulations that illustrate the robustness of our findings with respect to the model parameters.

In Fig. 8, we show that the exponent of the power-law used in the ADN mechanism has no impact on the qualitative behavior of the epidemic outbreaks. Our findings suggest that the parameter of the power-law does not change the qualitative behavior of the system, whereby children seem to be key drivers of the contagions when no specific policies are implemented. In fact, increasing the exponent of the power-law seems to have a moderate effect in decreasing the peaks of the outbreak (in particular for adults), but does not shift the curves. This observation allows us to conclude that our choice of setting it equal to $-2.09$, based on Aiello et al. (2001), does not reduce the generality of our numerical findings.

In Fig. 9, we show that the initial number of exposed individuals has no statistically significant impact on the total number of exposed individuals in the population. This observation allows us to conclude that our choice of initiating the system with 1% of exposed individuals has no impact on the output and, thus, on the results of our numerical analysis.

In Fig. 10, we simulate a simple dynamic vaccination procedure, in which a constant fraction of the population is vaccinated on a daily basis, until the total amount of population eligible for vaccination is reached, similar to Truszkowska et al. (2021). For the sake of simplicity, we assume that vaccinations start the same day in which the simulations start. The simulations are performed with the same parameters of Fig. 5 (that is, considering the original strain and the Alpha variant), and fixing the maximum fraction of the population eligible for vaccination to 60%. For low vaccination rates ($<0.5\%$ population/day), in both scenarios, the epidemic outbreak spreads faster than the vaccination campaign, resulting in a dramatic diffusion of the infection. On the contrary, we observe that if the vaccination speed is sufficiently fast (that is, above $0.5/1\%$ population/day), then the vaccination campaign is successful in protecting a large majority of the population, obtaining results that are consistent with those obtained in the simplified scenario of a static vaccination (Fig. 5). We further note that, once this critical threshold for the vaccination speed is reached (which is approximately equal to the peak speed of the vaccination campaign reached during spring 2021), we do not observe any remarkable benefit in further speeding up the vaccination process. The flexibility of our modeling framework allows to perform further analyses on the dynamic vaccination policies, including shifting the starting day of the vaccination campaign and considering non-constant vaccination speeds. We believe that these analyses, which are beyond the scope of this paper, could be pursued in future studies, toward better understanding the design of optimal vaccination policies during epidemic outbreaks.

The case study presented in this paper was calibrated to the situation in France during the spring 2021 COVID-19 pandemic, where the Alpha variant was quickly becoming dominant and replacing the original strain. At the end of spring 2021, the more infectious Delta variant (B 1.617.2) started spreading in most European countries and quickly became dominant as of the beginning of July 2021. In Fig. 11, we report the simulations performed in Fig. 6 for a fourth scenario, calibrated to the Delta variant of COVID-19. Specifically, in Liu and Rocklöv (2021) it was estimated that the Delta variant has a basic reproduction number of 5.08, with 95% confidence interval of [3.2, 8], yielding ${\bar{\lambda }}_{\alpha }\in [0.056,0.14]$, with an average of ${\bar{\lambda }}_{\alpha }=0.09$. The results of our simulations are qualitatively consistent with our other findings, further highlighting the importance of vaccination for highly infectious variants (as already observed in Scenario III, for the Alpha variant).

## Abbreviations

ADN: Activity-driven network
CI: Confidence interval
NPI: Non-pharmaceutical intervention
SEIR: Susceptible–exposed–infectious–removed
